# Analysis of *Helicobacter pylori* resistance in patients with different gastric diseases

**DOI:** 10.1038/s41598-024-55589-2

**Published:** 2024-02-28

**Authors:** Yongfu Shao, Yifan Lin, Ziyi Fang, Jianing Yan, Tuo Zheng, Guoliang Ye

**Affiliations:** 1 Health Science Center, Ningbo University, Ningbo, 315211 China; 2 Department of Gastroenterology, The First Affiliated Hospital of Ningbo University, Ningbo, 315020 China; 3 Department of Gastroenterology, Taizhou Central Hospital (Taizhou University Hospital), Taizhou, 318000 China; 4 Institute of Digestive Disease of Ningbo University, Ningbo, 315020 China

**Keywords:** *Helicobacter pylori*, Antibiotic resistance, Antimicrobial susceptibility test, Atrophic gastritis, Proton pump inhibitor, Clinical microbiology, Gastrointestinal diseases, Microbiota

## Abstract

*Helicobacter pylori* (*H. pylori*) resistance is the most important risk factor for eradication failure. However, in most regions, antibiotic resistance rates of *H. pylori* in patients with different types of gastric mucosal lesions are still unclear. An 8-year clinical retrospective cohort study involving 2847 patients was performed. In this study, we first summarized and compared the resistance status of *H. pylori* in different years, ages, sexes, and gastric diseases. The resistance profiles of amoxicillin (AMX), clarithromycin (CLR), levofloxacin (LVX) and furazolidone (FR) and their changing trends in the clinic were described. Then, multiple antibiotic resistance in different gastric diseases and years were described and compared. The relationship between proton pump inhibitor (PPI) medication history and antibiotic resistance in *H. pylori* was also explored. Finally, an antibiotic resistance risk model was constructed for clinical resistance risk prediction. The overall resistance rates of AMX, CLR, LVX and FR in gastric diseases were 8.18%, 38.11%, 43.98%, and 13.73%, respectively. The mono resistance, double resistance, triple resistance, and quadruple resistance rates were 30.17%, 25.96%, 6.46%, and 0.63%, respectively. Compared with the period from 2014 to 2016, the rates of mono-resistance and multiple resistance all showed relatively downward trends in the past 5 years. Factors including age, sex, type of gastric lesions and recent PPI treatment history are associated with the antibiotic resistance rate of *H. pylori*. Atrophic gastritis is an important clinical feature of high-risk antibiotic resistance in *H. pylori*-infected patients. Patients with atrophic gastritis have higher risk of resistant strains infection. In this study, our data provide the association between antibiotic resistance of *H. pylori* and gastritis pattern, which indicate the higher risk of resistant strain infection if the patients with atrophic gastritis, PPI history and older age.

## Introduction

*Helicobacter pylori* (*H. pylori*) is a common pathogenic microorganism for digestive system diseases, infecting nearly 50% of the population worldwide^1,2^. *H. pylori* infection causes persistent damage to the gastric mucosa, leading to glandular atrophy and intestinal metaplasia in the long run^3^. It is very necessary to eradicate *H. pylori* from the stomach, as it plays an important part in the pathogenesis of some gastric diseases, such as chronic gastritis, peptic ulcers, and gastric cancer^4–7^.

Classic therapeutic regimens that the combination of proton pump inhibitors (PPIs) and 2 antibiotics for *H. pylori* eradication have been used worldwide^8,9^. On this basis, regimens has successively derived bismuth-containing quadruple therapies, sequential therapy, reverse sequential therapy, hybrid therapy, concomitant therapy and vonoprazan based therapeutic regimens^10,11^. Successful eradication treatment in these regimens mainly depends on the sensitivity of *H. pylori* to drugs, and its increasing resistance is the main reason for eradication failure^12^. So, reviewing resistance to select suitable antibiotics in therapeutic regimen is important. More importantly, the antibiotic resistance rate of *H. pylori* vary greatly among different populations and regions and are affected by many factors^13,14^. Specifically, antibiotic resistance rates of *H. pylori* in patients with different types of gastric mucosal lesions are still unclear. Whether the type of gastric mucosal lesion is a factor or feature associated with *H. pylori* resistance remains unknown.

Therefore, an 8-year clinical retrospective cohort study involving 2847 patients was performed to mainly analyze the antibiotic resistance rate of *H. pylori* in patients with different types of gastric mucosal lesions. In this study, we first summarized and compared the resistance status of *H. pylori* in different years, ages, sexes, and gastric diseases. The resistance profiles of amoxicillin (AMX), clarithromycin (CLR), levofloxacin (LVX) and furazolidone (FR) and their changing trends in the clinic were described. Then, multiple antibiotic resistance in different gastric diseases and years were described and compared. The relationship between proton pump inhibitor (PPI) medication history and antibiotic resistance in *H. pylori* was also explored. Finally, nomograms were constructed to predict the individual risk of antibiotic resistance.

## Materials and methods

### Patients and inclusion criteria

This is a clinical retrospective study, targeting clinical patients undergoing gastroscopy for *H. pylori* culture and antimicrobial susceptibility tests, aimed at further evaluating the relationship between types of gastric mucosal lesions and *H. pylori* resistance. Patients and their medical history were obtained from the First Affiliated Hospital of Ningbo University. From 2014 to 2021, a total of 2847 patients (49.21% females) with the average age of 48.04 were ultimately included in this study. The diagnosis of gastric mucosal lesion is based on endoscopic findings and biopsy pathology. The inclusion criteria in this study were as follows: (1) patients were local residents who had detailed and complete medical records; (2) patients with a history of gastroscopy and positive *H. pylori* culture of the gastric mucosa; (3) patients with an antimicrobial susceptibility test of *H. pylori*, corresponding gastroscopy and gastroscopic biopsy pathology report; (4) no antibiotic use within 3 months before gastroscopy and drug susceptibility testing. This study was approved by the Human Research Ethics Committee of the First Affiliated Hospital of Ningbo University (No. KS20227019). All clinical procedures in accordance with relevant guidelines and regulations. Informed consent was obtained from all participants. All patients’ privacy was respected and protected.

### Gastroscopic biopsy and *H. pylori* culture

Patients in this study had same procedures and processes during past gastroscopy. Following evaluation by clinical doctors, endoscopic mucosal biopsy for pathological diagnosis was performed at the main lesion of the stomach, which collect one piece of tissue using biopsy forceps. Biopsy specimen was immersed in formalin solution for pathological examination. At the same time, two biopsy specimens for *H. pylori* culture were collected from the body and antrum of stomach, which were immediately stored in brain–heart infusion broth (Oxoid, Basingstoke, UK) with 5% glycerin and then transported to laboratory for culture. The biopsy method follows the “Standardized Guidelines for the Diagnosis and Treatment of Gastric Cancer” and the “Consensus on the Diagnosis and Treatment of *Helicobacter pylori* Infection in China”.

### Antimicrobial susceptibility testing

Antimicrobial susceptibility tests were determined by the agar dilution method. Resistance breakpoints were according to the European Committee on Antibiotic Susceptibility Testing (EUCAST) guidelines.

### Information traceability and grouping criteria

Medical history data were traced from the electronic medical record system of the hospital. Patients in this study were grouped according to their age, sex, gastric mucosal lesions, and PPI treatment history. The age group was divided according to the WHO standard. The diagnosis of gastric mucosal lesions is based on endoscopic findings and biopsy pathology. Patients who met the following conditions were considered to have a recent history of PPI treatment: (1) a history of taking PPIs within 3 months; (2) continuous medication for more than 1 week.

### Statistical analysis

Statistical analyses in this study were performed by using Statistical Product and Service Solutions (SPSS) 19.0 software. Chi-square (χ^2^) tests were used to evaluate the differences in antibiotic resistance rates among different groups. Minimal inhibitory concentration (MIC) values were assessed with Student’s t test or one-way analysis of variance (ANOVA). *P* < 0.05 was considered statistically significant.

Nomogram prediction models and figures were analyzed and drawn by the R software package “rms[6.4.0]”. The C-index was used to validate the predictive performance of the nomograms.

## Results

### Resistance rates of *H. pylori* strains between different years

As shown in Table 1, the overall resistance rates of AMX, CLR, LVX, and FR in gastric diseases were 8.18%, 38.11%, 43.98%, and 13.73%, respectively. From the perspective of time, the resistance rate of AMX in the last 5 years was much higher than that in 2014–2016 (*P* = 0.003), whereas LVX and FR showed downward trends (*P* < 0.001 and *P* < 0.001, respectively). Moreover, 36.78% of *H. pylori* strains were susceptible to all four tested antibiotics, and 33.05% of strains showed resistance to more than one antibiotic. There were 30.17% mono resistance, 25.96% double resistance, 6.46% triple resistance, and 0.63% quadruple resistance (Table 2). Compared with the past, multiple resistance rates in the past five years had a relatively downward trend. The no resistance strain increased from 24.06 to 41.31%, while the double resistance and triple resistance strain decreased from 32.89 and 8.96% to 23.49% and 5.57%, respectively.

**Table 1:** Resistance rates of *H. pylori* strains between different years.

Year	No. of patients	Antibiotics				MIC($\overline{x}$ ± *s*)			
		AMX	CLR	LVX	FR	AMX	CLR	LVX	FR
2014–2016	748	42 (5.61%)	287 (38.37%)	410 (54.81%)	230 (30.75%)	13.333 ± 4.647	14.034 ± 4.410	12.324 ± 10.289	8.265 ± 6.891
2017–2021	2099	191 (9.10%)	798 (38.02%)	842 (40.11%)	161 (7.67%)	6.658 ± 15.481	13.331 ± 12.021	12.295 ± 13.347	12.031 ± 12.940
Total	2847	233 (8.18%)	1085 (38.11%)	1252 (43.98%)	391 (13.73%)	7.862 ± 14.378	13.517 ± 10.558	12.305 ± 12.425	9.816 ± 10.001
*P*		0.003	0.865	< 0.001	< 0.001	0.006	0.333	0.969	0.001

**Table 2:** Multiple resistance rates between different years.

Patterns	2014–2016 (*n* = 748)	2017–2021 (*n* = 2099)	Total	*P*
No resistance	180 (24.06%)	867 (41.31%)	1047 (36.78%)	< 0.001
Mono resistance	248 (33.16%)	611 (29.11%)	859 (30.17%)	0.495
Double resistance	246 (32.89%)	493 (23.49%)	739 (25.96%)	< 0.001
Triple resistance	67 (8.96%)	117 (5.57%)	184 (6.46%)	0.001
Quadruple resistance	7 (0.93%)	11 (0.52%)	18 (0.63%)	0.222

### Resistance rates of *H. pylori* strains in patients with different gastric diseases

There were significant differences in the antibiotic resistance rates of AMX (*P* = 0.02), CLR (*P* = 0.011), LVX (*P* < 0.001) and FR (*P* < 0.001) among different gastric mucosal lesions (Table 3). Overall, the rate of antibiotic resistance in patients with atrophic gastritis was generally higher than that in other groups, which indicate patients with atrophic gastritis have higher risk of resistant strains infection (Table 3). In terms of double antibiotic resistance patterns, CLR and LVX were the most common combination (17.84%) in all gastric mucosal lesions, whereas the combination of AMX and FR (0.18%) had the lowest drug resistance rate (Table 4).

**Table 3:** Resistance rates of *H. pylori* strains in different gastric diseases.

Gastric diseases	No. of patients	Antibiotics				MIC ($\overline{x}$ ± *s*)			
		AMX	CLR	LVX	FR	AMX	CLR	LVX	FR
Superficial gastritis	558	47 (8.42%)	203 (36.38%)	179 (32.08%)	46 (8.24%)	8.516 ± 14.313	13.542 ± 10.799	12.413 ± 13.698	13.739 ± 16.014
Erosive gastritis	1166	90 (7.72%)	482 (41.34%)	549 (47.08%)	163 (13.98%)	8.183 ± 15.430	13.587 ± 10.423	12.172 ± 12.247	10.141 ± 9.967
Peptic ulcer	560	37 (6.61%)	183 (32.68%)	241 (43.04%)	72 (12.86%)	9.980 ± 17.321	13.487 ± 10.893	11.888 ± 12.370	8.681 ± 6.974
Atrophic gastritis	510	49 (9.61%)	198 (38.82%)	263 (51.57%)	103 (20.20%)	5.495 ± 10.881	13.152 ± 10.220	12.563 ± 11.405	7.495 ± 5.392
Intraepithelial neoplasia	53	10 (18.87%)	19 (35.85%)	20 (37.74%)	7 (13.21%)	5.650 ± 6.169	15.579 ± 12.357	16.600 ± 18.182	22.286 ± 20.377
*P*		0.02	0.011	< 0.001	< 0.001	0.646	0.912	0.584	< 0.001

**Table 4:** Patterns of multiple antibiotic resistance in different gastric diseases.

Antibiotics	Superficial gastritis (*n* = 558)	Erosive gastritis (*n* = 1166)	Peptic ulcer (*n* = 560)	Atrophic gastritis (*n* = 510)	Intraepithelial neoplasia (*n* = 53)	Total (*n* = 2847)
AMX + CLR	7 (1.25%)	17 (1.46%)	6 (1.07%)	6 (1.18%)	2 (3.77%)	38 (1.33%)
AMX + LVX	10 (1.79%)	14 (1.20%)	6 (1.07%)	5 (0.98%)	0 (0.00%)	35 (1.23%)
AMX + FR	3 (0.54%)	2 (0.17%)	0 (0.00%)	0 (0.00%)	0 (0.00%)	5 (0.18%)
CLR + LVX	77 (13.80%)	250 (21.44%)	86 (15.36%)	88 (17.25%)	7 (13.21%)	508 (17.84%)
CLR + FR	5 (0.90%)	28 (2.40%)	2 (0.36%)	9 (1.76%)	0 (0.00%)	44 (1.55%)
LVX + FR	11 (1.97%)	27 (2.32%)	25 (4.46%)	46 (9.02%)	0 (0.00%)	109 (3.83%)
AMX + CLR + LVX	11 (1.97%)	28 (2.40%)	9 (1.61%)	21 (4.12%)	2 (3.77%)	71 (2.49%)
AMX + CLR + FR	0 (0.00%)	4 (0.34%)	2 (0.36%)	1 (0.20%)	3 (5.66%)	10 (0.35%)
AMX + LVX + FR	2 (0.36%)	6 (0.51%)	6 (1.07%)	3 (0.59%)	0 (0.00%)	17 (0.60%)
CLR + LVX + FR	8 (1.43%)	25 (2.14%)	20 (3.57%)	32 (6.27%)	1 (1.89%)	86 (3.02%)
AMX + CLR + LVX + FR	4 (0.72%)	5 (0.43%)	2 (0.36%)	6 (1.18%)	1 (1.89%)	18 (0.63%)

### Resistance rates between different age groups and gender groups in different gastric diseases

We evaluated the effect of age and sex on the *H. pylori* strain resistance rate in different gastric diseases. LVX and AMX resistance rates were significantly different among different age groups (Table 5). After further subdivision of the data, we found that the LVX resistance rate in patients with superficial gastritis, erosive gastritis, peptic ulcer and atrophic gastritis was affected by age (*P* < 0.05), showing a high degree of consistency in different gastric diseases (Table 5). However, the resistance rates of AMX, CLR and FR only varied in some gastric mucosal lesions. Similar to the results in different age groups, CLR and LVX resistance rates in female group were significantly higher than those in male group (Table 6).

**Table 5:** Resistance rates of *H. pylori* strains between different age groups in different gastric diseases.

Antibiotics	Diseases	Age			*P*	MIC ($\overline{x}$ ± *s*)			*P*
		< 45	45–59	≥ 60		< 45	45–59	≥ 60	
AMX	Superficial gastritis	18/270 (6.67%)	17/206 (8.25%)	12/82 (14.63%)	0.075	5.306 ± 8.365	13.338 ± 20.986	6.500 ± 7.125	0.218
	Erosive gastritis	41/481 (8.52%)	32/480 (6.67%)	17/205 (8.29%)	0.528	8.787 ± 17.435	6.992 ± 12.787	8.971 ± 15.591	0.864
	Peptic ulcer	9/236 (3.81%)	12/216 (5.56%)	16/108 (14.81%)	0.001	5.917 ± 6.457	15.667 ± 23.513	8.000 ± 15.888	0.379
	Atrophic gastritis	5/97 (5.15%)	28/238 (11.76%)	16/175 (9.14%)	0.171	10.000 ± 6.000	6.938 ± 13.711	1.563 ± 1.940	0.181
	Intraepithelial neoplasia	0/9 (0%)	4/17 (23.53%)	6/27 (22.22%)	0.282	/	8.375 ± 8.807	3.833 ± 3.474	0.279
	Total	73/1093 (6.68%)	93/1157 (8.04%)	67/597 (11.22%)	0.005	7.658 ± 13.961	9.315 ± 16.352	6.067 ± 11.619	0.368
CLR	Superficial gastritis	84/270 (31.11%)	90/206 (43.69%)	29/82 (35.37%)	0.018	11.810 ± 10.001	14.456 ± 11.207	15.724 ± 11.361	0.136
	Erosive gastritis	186/481 (38.67%)	209/480 (43.54%)	87/205 (42.44%)	0.290	12.844 ± 10.334	14.234 ± 10.767	13.621 ± 9.681	0.417
	Peptic ulcer	70/236 (29.66%)	75/216 (34.72%)	38/108 (35.19%)	0.428	10.871 ± 8.614	14.842 ± 11.804	15.632 ± 12.053	0.035
	Atrophic gastritis	45/97 (46.39%)	89/238 (37.39%)	64/175 (36.57%)	0.233	13.978 ± 9.631	13.079 ± 9.714	12.672 ± 11.370	0.062
	Intraepithelial neoplasia	5/9 (55.56%)	7/17 (41.18%)	7/27 (25.93%)	0.236	9.600 ± 12.522	20.000 ± 12.000	15.429 ± 12.528	0.377
	Total	390/1093 (35.68%)	470/1157 (40.62%)	225/597 (37.68%)	0.053	12.357 ± 9.918	14.241 ± 10.857	14.018 ± 10.869	0.024
LVX	Superficial gastritis	66/270 (32.35%)	83/206 (40.29%)	30/82 (36.59%)	< 0.001	10.212 ± 12.180	12.651 ± 14.730	16.600 ± 13.278	0.103
	Erosive gastritis	190/481 (39.5%)	252/480 (52.50%)	107/205 (52.20%)	< 0.001	11.353 ± 12.658	11.569 ± 11.322	15.047 ± 13.259	0.025
	Peptic ulcer	87/236 (36.86%)	102/216 (47.22%)	52/108 (48.15%)	0.042	10.552 ± 10.391	12.990 ± 13.157	11.962 ± 13.774	0.403
	Atrophic gastritis	58/97 (59.79%)	129/238 (54.20%)	76/175 (43.43%)	0.019	11.378 ± 8.858	12.946 ± 12.021	13.000 ± 12.087	0.561
	Intraepithelial neoplasia	4/9 (44.44%)	7/17 (41.18%)	9/27 (33.33%)	0.786	21.000 ± 28.729	8.571 ± 5.381	20.889 ± 19.160	0.370
	Total	405/1093 (37.05%)	573/1157 (49.52%)	274/597 (45.90%)	< 0.001	11.059 ± 11.841	12.252 ± 12.295	14.255 ± 13.306	0.004
FR	Superficial gastritis	23/270 (8.52%)	16/206 (7.77%)	7/82 (8.54%)	0.952	14.783 ± 18.268	11.250 ± 9.147	16.000 ± 21.541	0.741
	Erosive gastritis	68/481 (14.14%)	62/480 (12.92%)	33/205 (16.01%)	0.542	9.074 ± 6.991	11.161 ± 12.443	10.424 ± 10.047	0.486
	Peptic ulcer	30/236 (12.71%)	33/216 (15.28%)	9/108 (8.33%)	0.212	10.367 ± 8.475	7.697 ± 5.834	6.667 ± 4.000	0.208
	Atrophic gastritis	36/97 (37.11%)	50/238 (21.01%)	17/175 (9.71%)	< 0.001	7.889 ± 5.533	7.280 ± 4.895	7.294 ± 6.669	0.865
	Intraepithelial neoplasia	3/9 (33.33%)	2/17 (11.76%)	2/27 (7.41%)	0.135	12.000 ± 6.928	36.000 ± 39.598	24.000 ± 11.314	0.517
	Total	160/1093 (14.64%)	163/1157 (14.09%)	68/597 (11.39%)	0.162	9.925 ± 9.591	9.583 ± 10.095	10.118 ± 10.828	0.919

**Table 6:** Resistance rates of *H. pylori* strains between male and female in different gastric diseases.

Antibiotics	Diseases	Gender		*P*	MIC ($\overline{x}$ ± *s*)			*P*
		Male	Female		Male	Female	Total	
AMX	Superficial gastritis	25/238 (10.5%)	22/320 (6.88%)	0.127	6.443 ± 13.913	10.340 ± 14.694	8.516 ± 14.313	0.357
	Erosive gastritis	40/539 (7.42%)	50/627(7.97%)	0.724	7.331 ± 14.699	8.865 ± 16.105	8.183 ± 15.430	0.642
	Peptic ulcer	28/372 (7.53%)	9/188 (4.79%)	0.218	7.446 ± 16.444	17.861 ± 18.578	9.980 ± 17.321	0.118
	Atrophic gastritis	12/219 (5.48%)	37/291 (12.71%)	0.006	8.479 ± 19.682	4.527 ± 5.963	5.495 ± 10.881	0.279
	Intraepithelial neoplasia	4/33 (12.12%)	6/20 (30.00%)	0.107	2.875 ± 3.473	7.500 ± 7.141	5.650 ± 6.169	0.269
	Total	109/1401 (7.78%)	124/1446 (8.58%)	0.439	7.728 ± 13.518	8.014 ± 15.359	7.862 ± 14.378	0.439
CLR	Superficial gastritis	78/238 (32.77%)	125/320 (39.06%)	0.127	14.718 ± 10.534	12.808 ± 10.939	13.542 ± 10.799	0.221
	Erosive gastritis	209/539 (38.78%)	273/627 (43.54%)	0.099	14.077 ± 11.039	13.212 ± 9.929	13.587 ± 10.423	0.367
	Peptic ulcer	111/372 (29.84%)	72/188 (38.3%)	0.044	12.307 ± 10.842	15.306 ± 10.795	13.487 ± 10.893	0.069
	Atrophic gastritis	83/219 (37.90%)	115/291 (39.52%)	0.710	12.843 ± 9.653	13.374 ± 10.646	13.152 ± 10.220	0.720
	Intraepithelial neoplasia	9/33 (27.27%)	10/20 (50.00%)	0.094	20.889 ± 13.679	10.800 ± 9.247	15.579 ± 12.357	0.074
	Total	490/1401 (34.98%)	595/1446 (41.15%)	0.001	13.371 ± 10.380	13.694 ± 10.778	13.517 ± 10.558	0.616
LVX	Superficial gastritis	64/238 (26.89%)	115/320 (35.94%)	< 0.001	12.078 ± 14.142	12.600 ± 13.504	12.413 ± 13.698	0.808
	Erosive gastritis	232/539 (43.04%)	317/627 (50.56%)	0.010	11.795 ± 11.880	12.448 ± 12.531	12.172 ± 12.247	0.538
	Peptic ulcer	154/372 (41.40%)	87/188 (46.28%)	0.271	10.279 ± 10.520	14.736 ± 14.742	11.888 ± 12.370	0.007
	Atrophic gastritis	97/219 (44.29%)	166/291 (57.04%)	0.004	14.144 ± 11.609	11.639 ± 11.215	12.563 ± 11.405	0.086
	Intraepithelial neoplasia	10/33 (30.30%)	10/20 (50.00%)	0.152	12.400 ± 10.906	20.800 ± 23.232	16.600 ± 18.182	0.314
	Total	557/1401 (39.76%)	695/1446 (48.06%)	< 0.001	12.686 ± 12.920	11.829 ± 11.771	12.305 ± 12.425	0.225
FR	Superficial gastritis	17/238 (7.14%)	29/320 (9.06%)	0.415	12.235 ± 15.262	14.621 ± 16.640	13.739 ± 16.014	0.631
	Erosive gastritis	80/539 (14.84%)	83 (13.24%)	0.431	12.750 ± 9.580	9.554 ± 10.350	10.141 ± 9.967	0.446
	Peptic ulcer	46/372 (12.37%)	26/188 (13.83%)	0.625	8.826 ± 7.523	8.423 ± 6.014	8.681 ± 6.974	0.816
	Atrophic gastritis	48/219 (21.92%)	55/291 (18.90%)	0.401	7.750 ± 5.973	7.272 ± 4.874	7.495 ± 5.392	0.656
	Intraepithelial neoplasia	2/33 (6.06%)	5/20 (25.00%)	0.048	16.000 ± 0	24.800 ± 24.397	22.286 ± 20.377	0.650
	Total	193/1401 (13.78%)	198/1446 (13.69%)	0.949	9.899 ± 10.877	9.731 ± 9.042	9.816 ± 10.001	0.868

### Effect of PPI treatment history on antibiotic resistance rate

PPI treatment history was also a factor affecting *H. pylori* resistance rates. As shown in Table 7, the resistance rates of AMX, CLR and LVX in patients who recently took PPI in three months were 11.28%, 47.18% and 47.86%, respectively, which were all significantly higher than those in patients without PPI treatment history. However, the opposite trend was observed in FR under the same circumstances. It is observed that a recent PPI treatment history will increase the antibiotic resistance rate by 4%-12%, except for FR.

**Table 7:** Effect of PPI treatment history on antibiotic resistance rate.

History of PPI treatment	No. of patients	Antibiotics				MIC ($\overline{x}$ ± *s*)			
		AMX	CLR	LVX	FR	AMX	CLR	LVX	FR
No PPI history	2262	167 (7.38%)	809 (35.76%)	972 (42.97%)	329	7.488 ± 13.613	12.872 ± 9.845	12.498 ± 12.311	9.693 ± 9.641
PPI history	585	66 (11.28%)	276 (47.18%)	542 (47.86%)	62 (10.06%)	8.807 ± 16.227	15.410 ± 12.240	11.634 ± 12.810	10.468 ± 11.796
*P*		0.002	< 0.001	0.034	0.013	0.529	< 0.001	0.305	0.576

### Nomogram development for antibiotic resistance risk

**Figure 1:**
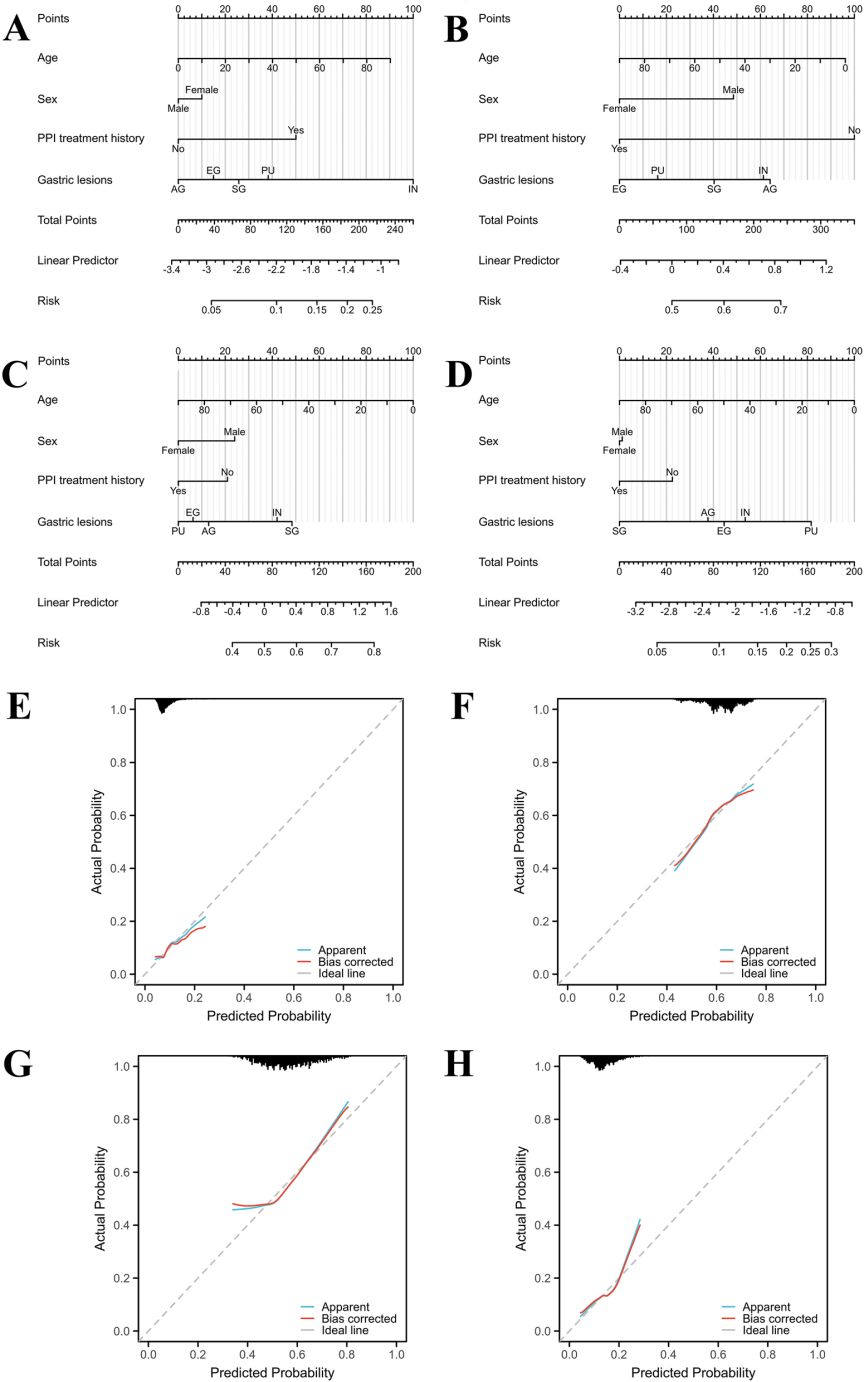
Nomogram development for antibiotic resistance risk. Nomogram models were constructed to predict the risk of amoxicillin (**A**), clarithromycin (**B**), levofloxacin (**C**) and furazolidone (**D**). Calibration curves reflected good discriminative ability of models for amoxicillin (**E**), clarithromycin (**F**), levofloxacin (**G**) and furazolidone (**H**). Individual factors such as age, sex, PPI treatment history and gastric disease are all incorporated into antibiotic resistance prediction models. In clinical practice, users can calculate total points of the model based on individual factors, and then conveniently obtain the probability data of specific antibiotic resistance to predict the individual risk. For example, a 45 year-old man with PPI history and his endoscopic finding was atrophic gastritis, the risk of clarithromycin resistance will exceed 60%. Therefore, clarithromycin should not be prioritized for use, and bismuth-containing quadruple therapy should be considered as the first line empirical treatment for this patient according to Maastricht consensus. SG, superficial gastritis; EG, erosive gastritis; PU, peptic ulcer; AG, atrophic gastritis; IN, intraepithelial neoplasia.

Age, sex, PPI treatment history and gastric disease were all incorporated into nomograms to predict the individual risk of antibiotic resistance (Fig. 1). The C-index of the nomograms for AMX, CLR, LVX and FR was 0.592, 0.578, 0.609, and 0.601, respectively. Calibration curves reflected good discriminative ability (Fig. 1).

## Discussion

Currently, eradication of *H. pylori* faces a series of challenges. The increasing proportion of antibiotic-resistant strains and the emergence of multidrug-resistant strains have increased the difficulty of antibiotic selection^15–17^. For example, AMX is widely used as a first-line treatment in current therapeutic regimens for *H. pylori* eradication due to its affordability and safety^11^. However, due to the widespread emergence of AMX-resistance strains, amoxicillin in Africa has lost its clinical application value, in where the overall rate of resistance to AMX have reached as high as 72.6%^18^. In addition, the lack of sufficient retrospective studies to provide guidance for eradication therapy and the differences in resistance among different populations and regions also make the success rate of primary eradication unsatisfactory^19,20^. In the absence of an antibiotic sensitivity test, rational selection of antibiotics has become the key to *H. pylori* eradication therapy.

To change this situation and increase the rationality of antibiotic selection, we need to conduct a retrospective study on the resistance of commonly used antibiotics. Since AMX, CLR, LVX, and FR are commonly used antibiotics in *H. pylori* eradication therapy in China^21,22^, in this study, an 8-year clinical retrospective study was performed to mainly analyze the antibiotic resistance rate of *H. pylori* in patients with different types of gastric mucosal lesions. Our statistical results showed that the overall resistance rates of AMX, CLR, LVX, and FR were 8.18%, 38.11%, 43.98% and 13.73%, respectively. The resistance rates of CLR and LVX were greater than 30%, which implies that they were not suitable for empirical treatment unless under the guidance of an antimicrobial sensitivity test. Among all the types of double resistance patterns, CLR and LVX were the most common combination in all gastric mucosal lesions, whereas the combination of AMX and FR had the lowest drug resistance rate, which suggests that AMX + FR is the best combination for *H. pylori* eradication therapy, especially in the first empirical treatment. Moreover, compared with the period from 2014 to 2016, the rates of mono-resistance and multiple resistance all showed relatively downward trends in recent 5 years (Table 2). Within the region, the no resistance strain increased from 24.06 to 41.31%, while the double resistance and triple resistance strain decreased from 32.89% and 8.96% to 23.49% and 5.57%, respectively. This situation may be owing to the relatively strict management of antibiotic application in China in recent years^23^. However, it is worth noting that compared with neighboring countries, our *H. pylori* antibiotic resistance rate is still at a relatively high level^24–26^.

*H. pylori* infection causes different types of gastric mucosal lesions, and it remains unclear whether the type of gastric mucosal lesion a factor or feature associated with *H. pylori* resistance. There is no relevant research report on this issue at present. Therefore, the relationship between the type of gastric lesions and the *H. pylori* resistance rate is the theme of this study. Remarkably, according to our data, we confirmed that there are significant differences in the resistance rates of these four antibiotics among different gastric mucosal lesions. More specifically, patients with atrophic gastritis have more serious *H. pylori* resistance than other groups. In the atrophic gastritis group, the resistance rates of the four antibiotics were all higher than the average level. Patients with atrophic gastritis have higher risk of resistant strains infection. Altogether, our results indicated that atrophic gastritis is an important clinical feature of high-risk antibiotic resistance in *H. pylori*-infected patients. This phenomenon may be related to bacterial-specific virulence factors such as cytotoxin-associated gene A (CagA) and vacuolating cytotoxin A (VacA). For example, CagA is an important effector protein of *H. pylori*, which was previously demonstrated to not only activate cell senescence and cause gastric mucosal atrophy but also promote *H. pylori* strains to further form biofilms, thus causing multidrug resistance^27–29^.

The antibiotic resistance rate of *H. pylori* vary greatly among different populations and regions and are affected by many factors, such as age, sex and medication history^13,30^. In our study, we found that the resistance rates of CLR and LVX in females were significantly higher than those in males. The resistance rates of AMX and LVX in different age groups were significantly different. Our results suggest that the distribution of antibiotic resistance was associated with age and sex. Therefore, we should consider the patient's age and sex when choosing antibiotics for *H. pylori* eradication.

It is worth mentioning that it remains unclear whether PPI treatment history before eradication therapy will affect the resistance rate of *H. pylori*. Previous studies have suggested that PPIs cause *H. pylori to* migrate from the antrum to the gastric body and transform into a spherical shape, thereby reducing the sensitivity of *H. pylori* to antibiotics^31,32^. However, some meta-analyses revealed that preadministration of PPIs did not affect the final eradication rate of *H. pylori* infection^33,34^, suggesting that PPI treatment history before eradication therapy may not affect *H. pylori* resistance. Thus, we assessed the effect of PPI treatment history on the antibiotic resistance rate in this study. We observed that a recent PPI treatment history will increase the antibiotic resistance rate by 4–12%, except for FR, hinting at multiple mechanisms for PPIs in response to antibiotic resistance.

As the proportion of antibiotic-resistant *H. pylori* strains continues to rise and the emergence of multidrug resistance strains, eradication therapy are constantly evolving^11^. Due to the declining efficacy of legacy triple therapies, bismuth-containing quadruple therapies have been recommend as the best initial empiric treatment^35^. Some bismuth-free quadruple options such as concomitant, sequential and hybrid therapies are only recommended for areas with low clarithromycin and metronidazole resistance^35^. Moreover, vonoprazan-containing regimens show high efficacy in terms of *H. pylori* eradication compared with PPI-containing therapy, which indicate future therapies may be influenced by adding the novel potassium-competitive acid blocker^11,36,37^. However, comprehensive consideration of local antibiotic resistance and individual factors is necessary to propose more effective individualized therapeutic regimens. In this study, we established antibiotic resistance prediction models based on individual factors such as age, sex, PPI treatment history and gastric disease, which all closely related to *H. pylori* resistance. Our models are presented in the form of nomograms, which will be better used by clinical doctors for predicting patients’ individual risk of antibiotic resistance.

Admittedly, our research has some limitations. The major limitations of the present research is that it is a single center study with a single source of patient population. Moreover, our study is a retrospective study. Although most patients in this study have no previous history of *H. pylori* eradication treatment, there are still a very small number of patients who have undergone eradication failure. In addition, our study is based on the results of antimicrobial susceptibility testing in vitro, and the potential risk of heteroresistance of *H. pylori* should not be ignored^38,39^. However, these limitations have extremely limited influence and do not affect the credibility of our study.

In summary, our data provide the association between antibiotic resistance of *H. pylori* and gastritis pattern, which indicate factors including age, sex, type of gastric lesions and recent PPI treatment history are associated with *H. pylori* resistance rate. Atrophic gastritis is an important clinical feature of high-risk antibiotic resistance in *H. pylori*-infected patients. Patients with atrophic gastritis, PPI history and older age have higher risk of resistant strains infection.

## Data Availability

The data that support the findings of this study are available from the corresponding author upon reasonable request.
